# Time scale of resilience loss: Implications for managing critical transitions in water quality

**DOI:** 10.1371/journal.pone.0223366

**Published:** 2019-10-07

**Authors:** Ryan D. Batt, Tarsha Eason, Ahjond Garmestani

**Affiliations:** 1 National Research Council, United States Environmental Protection Agency, Cincinnati, Ohio, United States of America; 2 Rensselaer Polytechnic Institute, Department of Biological Sciences, Troy, New York, United States of America; 3 Rutgers University, Department of Ecology, Evolution, and Natural Resources, New Brunswick, New Jersey, United States of America; 4 United States Environmental Protection Agency, Office of Research and Development, Research Triangle Park, North Carolina, United States of America; 5 United States Environmental Protection Agency, Office of Research and Development, Cincinnati, Ohio, United States of America; 6 Utrecht Centre for Water, Oceans and Sustainability Law, Utrecht University School of Law, Utrecht, Netherlands; Swedish University of Agricultural Science, SWEDEN

## Abstract

Regime shifts involving critical transitions are a type of rapid ecological change that are difficult to predict, but may be preceded by decreases in resilience. Time series statistics like lag-1 autocorrelation may be useful for anticipating resilience declines; however, more study is needed to determine whether the dynamics of autocorrelation depend on the resolution of the time series being analyzed, i.e., whether they are time-scale dependent. Here, we examined timeseries simulated from a lake eutrophication model and gathered from field measurements. The field study involved collecting high frequency chlorophyll fluorescence data from an unmanipulated reference lake and a second lake undergoing experimental fertilization to induce a critical transition in the form of an algal bloom. As part of the experiment, the fertilization was halted in response to detected early warnings of the algal bloom identified by increased autocorrelation. We tested these datasets for time-scale dependence in the dynamics of lag-1 autocorrelation and found that in both the simulation and field experiment, the dynamics of autocorrelation were similar across time scales. In the simulated time series, autocorrelation increased exponentially approaching algal bloom development, and in the field experiment, the difference in autocorrelation between the manipulated and reference lakes increased sharply. These results suggest that, as an early warning indicator, autocorrelation may be robust to the time scale of the analysis. Given that a time scale can be shortened by increasing sampling frequency, or lengthened by aggregating data during analysis, these results have important implications for management as they demonstrate the potential for detecting early warning signals over a wide range of monitoring frequencies and without requiring analysts to make situation-specific decisions regarding aggregation. Such an outcome provides promise that data collection procedures, especially by automated sensors, may be used to monitor and manage ecosystem resilience without the need for strict attention to time scale.

## Introduction

Regime shifts are abrupt changes that arise when an ecosystem exhibits a nonlinear or discontinuous response to a driver variable causing it to undergo radical change due to an incremental increase in external forcing. Examples of regime shifts include transitions from clear to turbid water with increased nutrient loading to lakes [1], forests to prairies with increased fire [2–4], or collapses of fisheries with increased harvest [3, 5, 6]. It is often impossible to restore ecosystems to pre-shift conditions, and even transient losses of ecosystem function can have negative consequences for humans and nature. Regime shifts are notoriously difficult to predict because the critical value of the driver variable (nutrients, fires, or harvest) at which the ecosystem undergoes a dramatic reconfiguration is difficult to estimate from data and varies among ecosystems and time periods, even if conditions seem similar. The difficulty of predicting regime shifts is a major impediment to ecosystem management.

Instead of trying to predict regime shifts by estimating critical values, critical slowing down results in generic changes in dynamical behavior that can be used to signal the approach of a critical transition. When an ecosystem approaches a critical transition, its dynamics “slow down”, resulting in increases in summary statistics like variance and autocorrelation [7, 8]. This slowing down occurs because an ecosystem near a critical transition has low resilience, resulting in a slower approach to equilibrium following perturbation [9]. This behavior is theorized to be generic, and therefore forecasts of regime shifts based on critical slowing down are not subject to the same problem of system specificity that complicates other approaches. Early warning signals based on critical slowing down have been shown to presage critical transitions in models [9–11], laboratory experiments [12–14], historical time series [7, 15, 16] and in ecosystem experiments [17, 18]. Though in its infancy, the success of early warning signals in forecasting regime shifts in these examples suggests promise for the approach in an applied setting.

Much of the research community’s excitement about early warning signals is due to their potential for use in management. To this end, substantial research has been conducted on topics of both theoretical and practical importance, such as what types of transitions may be preceded by early warnings [15, 19, 20], and whether early warnings are discernable prospectively, not just retrospectively [21, 22]. The development and assessment of statistics suitable for detecting critical slowing down has been an especially active topic of research [23, 24]. Unfortunately, most of these statistics are data-hungry, requiring time series of substantial duration and resolution. However, this demand can be readily met with autonomous systems, such as remote sensing by satellites [25] or *in situ* sampling by automated sensors [26]. Automated sensors, especially *in situ* devices, remove traditional barriers to high frequency sampling, and are routinely used by researchers to produce time series with resolutions of minutes to fractions of a second [27, 28]. Furthermore, such devices are used by the Global Lake Ecological Observatory Network, the Long-Term Ecological Research program, the National Ecological Observatory Network, the Ocean Observatory Initiative, and federal agencies like NASA, NOAA, USGS, and EPA, which, along with individual researchers, are making the availability and sharing of large quantities of high frequency data more commonplace [29]. Thus, high-frequency data streams will likely become central to computing early warning statistics, especially in the context of ecosystem management.

Time scale can be influenced by sampling frequency and by downsampling or aggregating a time series. Time scale can never be shorter than the frequency of measurements, but frequent measurements can be made more coarse during analysis (until a time series’ resolution equals its extent). Thus, analysts of high-frequency time series are faced with the choice of analyzing at their native time scale (i.e., “as-is”), or downsampling the data to longer time scales. Downsampling choses a subset of the data and lengthens the time scale because it increases the amount of time between values in the time series. Many ecological processes are well-known to be scale dependent [30], which means that sampling or analysis choices affecting time scale might determine whether early warning signals are present, muted, or absent. To date, the consequence of choice of time scale on early warning statistics has rarely been tested, and only in a model setting [31], where it was suggested that statistics were robust to time scale. Ambiguous or conflicting statistical results can undermine the applied utility of early warning signals because management needs to be based on clearly defined procedures and conditions [32]. When subjective or fuzzy decisions lead to alternate recommendations, this ambiguity undermines the authority of the warning system and makes the system unlikely to prompt intervention or other calls to action [20]. Furthermore, the performance of a warning system should be validated in both theoretical and field settings to give the system the greatest amount of credibility. Therefore, determining whether common early warning statistics are time-scale dependent in a field setting is a critical step towards their application.

A commonly used early warning statistic is autocorrelation. In this context, autocorrelation is almost exclusively calculated as first-order (or “lag-1”) autocorrelation, which is the correlation between adjacent observations in a time series. However, adjacent observations could be separated by a century or a second. The first study to use autocorrelation as an early warning indicator applied it to output from a model of ocean thermohaline circulation after aggregating the resulting time series to 50-year time steps, and found that autocorrelation increased towards 1 as the bifurcation was approached [10]. Several cross-scale statistics have also been developed. Detrended fluctuation analysis (DFA) has been used as an early warning indicator of a critical transition in an oceanographic model, and involves analyzing changes in the exponent of a power law relating log-autocorrelation to log-time scale [11]. While this approach incorporates information from multiple time scales, it 1) assumes the presence of a power law relationship, and 2) does not specifically address whether or not the autocorrelation at any single time scale increases towards 1, as would be expected if it were to signal an approaching bifurcation. Similarly, the spectral ratio is an early warning indicator that signals a bifurcation when more power shifts to low frequencies relative to high frequencies [21, 33, 34]. The variance spectrum can be estimated as the Fourier transform of the autocorrelation function; however, as with DFA, the temporal pattern of a constituent time scale cannot be inferred from the ratio. Both DFA and spectral ratio may be useful early warning indicators, but they are not used as commonly as lag-1 autocorrelation, which has been applied at time scales varying by several orders of magnitude. High frequency data can be downsampled to lengthen the time scale of an analysis, but this decision is typically made with expert knowledge of the system’s dynamics and to increase analytical tractability [18, 26]. It is unclear whether similar results would have been found if autocorrelation was analyzed at different time scales.

In lakes, several types of bifurcations are known to be associated with increased nutrient load, including turbidity in shallow lakes [1] and eutrophication in deep lakes [35]. The eutrophication of a deep lake involves abrupt changes in the exchange of phosphorus between sediment and water, with high nutrient loading leading to high water phosphorus, and, implicitly, low water quality due to high algal biomass [36, 37]. The critical transition from low water P to high water P is abrupt: a small change in P loading causes the system to undergo a saddle-node (fold) bifurcation. In the related but distinct context of planktonic interactions, increased nutrient loading can result in a Hopf bifurcation, another type of critical transition, wherein a stable equilibrium gives way to stable oscillations [38–40]. The state variables involved in this transition are unicellular algae, colonial algae, and herbivorous zooplankton. At low nutrients, colonial algae are rare, and there are no oscillations. As nutrient loading increases to a critical level, colonial algae begin to dominate the algal community and all three state variables begin to oscillate. In simulation models, this critical transition from a stable point to a stable limit cycle is preceded by increasing variance and autocorrelation [41].

The goal of this paper is to test whether the dynamics of autocorrelation are time-scale dependent. We begin with the relatively simple case of the mathematical model of a lake undergoing eutrophication and a saddle-node bifurcation. Critical slowing down is well-studied for saddle-node bifurcations, and these bifurcations are common in ecology. Next, we test for time-scale dependence in field data collected during an experimental lake fertilization designed to induce a bloom of colonial algae; this scenario resembles theoretical systems involving a Hopf bifurcation. Though distinct, both scenarios involve a critical transition in water quality. Previous analyses of the field experiment found early warning signals in autocorrelation [18, 42]. Although these studies analyzed manually-sampled chlorophyll and automated measures of phycocyanin, the present study is the first to analyze automated measurements of chlorophyll from this experiment. Algal blooms are a management concern throughout the world, and have been receiving increased attention in the freshwater ecosystems in the United States. Effective warning systems for algal blooms require clear guidance on implementation. Because time scale influences implementation for both field sampling and data analysis, our results provide information key to designing an effective approach to monitoring for early warning signals of critical transitions.

## Materials and methods

### Mathematical model and simulation experiments

The mathematical model of phosphorus dynamics in lake water (*X*, g P m^-2^) and lake sediment (*M*, g P m^-2^) has the following deterministic skeleton:

$$\frac{dX}{dt}=I-X(s+h)+rMR$$ (1)

$$\frac{dM}{dt}=sX-bM-rMR$$ (2)

$$R=\frac{X^{q}}{m^{q}+X^{q}}$$ (3)

The system gains phosphorus through input from the watershed (*I*, g P m^-2^ y^-1^) to the water, and loses P through outflow from *X* (controlled by the constant *h* = 0.15 y^-1^) and through permanent burial of *M* (controlled by the constant *b* = 0.001 y^-1^). Sedimentation of *X* (the product of *X* and *s* = 0.7 y^-1^) transfers P from *X* to *M*. The last term of Eqs 1 and 2 describes recycling of P from *M* to *X*, with the maximum recycling rate being controlled by the constant *r* (0.019 y^-1^). In Eq 3, R describes the portion of the recycling term controlled by water P and the constant *m* (2.4 g m^-2^) determines the value of *X* when the recycling is at half of *r*. Total recycling (rMR) is also governed by r (a constant rate-controlling value) and M (sediment P) (Eq 2). The exponent *q* (8, unitless) controls the steepness of the of the sigmoidal function when *X = m*. At low *X*, minimal P is transferred from *M* to *X*, whereas at high *X* this flux is much larger. With the exception of simplifying P input into a single controllable variable (*I*), the model structure and parameterization follows that of [43], who used the model to study variance as an indicator of critical slowing down.

**Fig 1:**
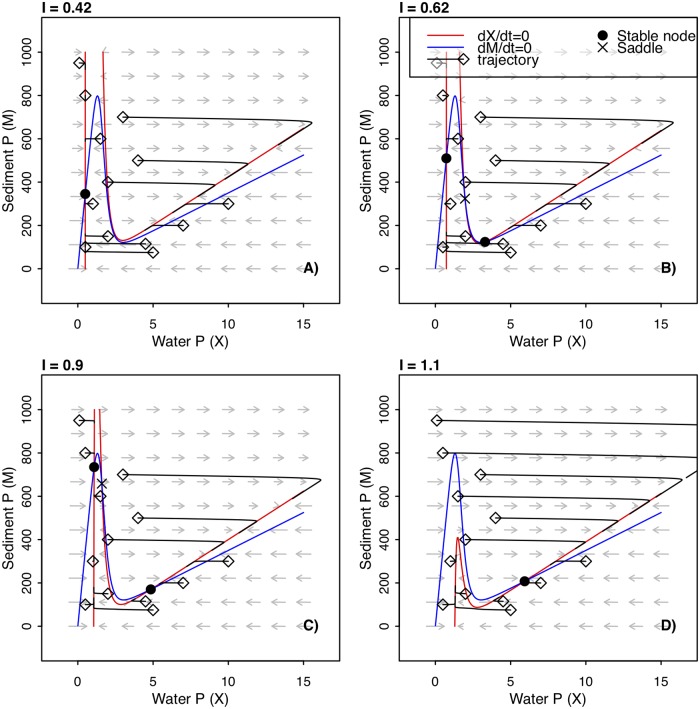
A phase portrait of the model system for varying values of P input. The vector field is represented by gray arrows. Nullclines are represented by red and blue lines, indicating where dX/dt and dM/dt are equal to zero, respectively. Trajectories starting at arbitrary initial points (open diamonds) and continuing along the accompanying solid black line indicate how the system moves from the initial point through phase space for 50 years. Equilibria are indicated by points: solid filled circle is a stable node, an ‘X’ is a saddle point. An equilibrium occurs where the nullclines cross. The different panels correspond to different values of P loading (*I*).

We used the model in Eqs 1–3 to generate changes in autocorrelation via critical slowing down. To produce critical slowing down, we induced a bifurcation by manipulating *I*, the rate of P input. The critical points of *I* were at approximately 0.5195 and 0.9976 g P m^-2^ y^-1^. At *I* < 0.5195, a single equilibrium value exists: a stable node at low *X* and high *M* (Fig 1a). As *I* increases to the first critical value, a second stable node emerges at high *X* and low *M*, as well as an unstable equilibrium (saddle point) that separates the two stable nodes (Fig 1b). As *I* continues to increase, the first stable node at low *X* and high *M* nears the saddle point (Fig 1c), until they collide and annihilate each other at the second critical value of *I*. At *I* > 0.9976, the only equilibrium value is the stable node at high *X* and low *M* (Fig 1d); therefore, the second critical value is where the saddle-node bifurcation occurs, as the stable state of high water quality (low *X*) disappears and the system transitions to a stable state of low water quality (high *X*).

We performed two sets of stochastic simulations based on Eqs 1–3. In the first simulation we increased *I* in a stepwise fashion; for each tier of *I*, the state variables were initiated at their equilibrium values (if bistability was present, equilibrium was set to the stable node with the lower value of *X*), and then the stochastic simulation proceeded for 200 years with a time step of *dt* = 1/24. To make the model stochastic, we added the independent noise terms *σ*_*X*_*ε*_*X*,*t*_, *σ*_*M*_*ε*_*M*,*t*_ (both *ε*~*N*(0,1)) to Eqs 1 and 2, respectively. In all simulations, *σ*_*X*_ = 0.005 and *σ*_*m*_ = 0.1. We used 50 tiers of *I* which formed an evenly spaced sequence beginning at *I* = 0.419 in the first tier, and ending at *I* = 1.098 in the final tier. The second simulation used the same time step size and range of *I* values as the first, but in this case the simulation lasted 200 years, *I* was incremented each time step, and the system was only set to equilibrium at the first time step. Furthermore, in addition to the stochastic terms used in the first simulation, we added innovations that integrated to a sine wave with a period of one year and amplitude α; i.e., *α* cos(2*πt*)2*π* (*α*_*X*_ = 0.0075 *and α*_*M*_ = 0.005). The sine wave mimics cyclic patterns that occur in many natural systems (e.g., daily or annual time scales), which may need to be filtered out to achieve stationarity before estimating autocorrelation. Our statistical analysis of the simulations will be focused on time series of *X*, a variable commonly measured by limnologists.

### Description of experimental lakes

Our empirical time series consist of fluorescence data measured at 5-min intervals from May 9, 2015 (day of year 129) to September 4, 2015 (day of year 247; 119 days total) in two lakes, Peter Lake and Paul Lake, during a whole-lake experiment that tested for early warning signals of algal blooms [18]. Peter Lake and Paul Lake are small (2.7 ha and 1.7 ha, respectively), deep (max depth = and 19.9 m and 12.8 m, respectively) lakes located at the University of Notre Dame Environmental Research Center in the upper peninsula of Michigan, USA (89°32’ W, 46°13’ N). Peter Lake and Paul Lake were originally two basins of a single hourglass-shaped lake until they were divided by an earthen dike for the purpose of limnological experiments in which Paul Lake has served as a reference system because its waters flow into Peter Lake [44]. The lakes stratify during the summer (e.g., in 2015, depth of epilimnion = 2–4 m), and are typically low-productivity (total phosphorus = 10–15 μg/L, chlorophyll = 5–10 μg/L) with clear but slightly brown water (Secchi = 4–5 m, DOC = 4–6 mg/L, depth of 1% surface irradiance = 4–6 m) [18, 45]. In 2015, an experiment was conducted that involved fertilizing Peter Lake in order to induce a critical transition in the form of an algal bloom. The experimental fertilization of Peter Lake began on day 152 by adding a solution of H_3_PO_4_ and NH_4_NO_3_ to its surface water each day, at a P loading rate of 3 mg / m^2^ / d and N:P molar ratio of 15:1. Fertilization of Peter Lake ceased on day 180 in response to the detection of early warning signals [18].

### Empirical time series

The high frequency measurements were made by fluorometer probes on board sondes, which are automated *in situ* sensors. Two sondes were present in each lake: one YSI V2–6600 multiparameter sonde, and one Hydrolab DS5X. The fluorescence probes measured chlorophyll *a* (YSI model 6025, Hydrolab model 007291). Sensor faces were manually cleaned once a day, and calibrated once a week according to manufacturer recommendations. Two sondes were used in each lake so that data gaps in one time series (e.g., due to sensor removal for calibration) could be filled with observations made by the other sensor. Because measurements made by one sensor might be higher or lower than those made by the other, we used a multivariate autoregressive state space (MARSS) model to calibrate the two time series to each other [46]. In effect, values in each time series were predicted by that time series’ history and the history of the second time series. Our analysis focuses on measurements made by the Hydrolab instrument at a depth of 0.5 m, and we used MARSS estimates to impute missing values.

### Data analysis—Transformation, stationarity

To remove nonstationarities prior to calculating autocorrelation, the time series from the second simulation was detrended, and the empirical time series were detrended and log-transformed. For analyses involving aggregation, detrending was performed after aggregating (see below). We took the natural logarithm of the time series in order to stabilize their variance. We detrended the time series by fitting a regression model based on three sets of predictor variables. The mean and possible linear trend were removed with a polynomial of maximum order *P = 1*. For a *P*-order polynomial and a time index *t* = 1, 2, … *n*, where *n* is the length of the time series, there would be *P+1* vectors (*t*^*P* = 0^, *t*^*P* = 1^) as predictor variables. The second set of predictor variables contained a *K*-order Fourier series of *K* pairs of sine and cosine waves with a nominal period (for the first pair) of 24 hours for the empirical series and 1 year for the simulated series, such that the *k*-th sine-cosine pair at time *t* would be $\text{sin}(\frac{2\pi kt}{m})$ and $\text{cos}(\frac{2\pi kt}{m})$, where *m* is the number of observations per period (for 5-minute data, *m* = 288). The purpose of the Fourier series was to capture the strong daily cycle present in *in situ* fluorescence data or the sine wave added to the simulated time series. The third set of predictors consisted of an *I*-th order interaction between the polynomial trend and the Fourier series, resulting in I*2K interaction vectors. The interaction would account for a trend in the amplitude of the cycle. Thus, the complete model takes the form

$$\begin{array}{l} y_{t}=\sum_{p=0}^{p=P}\left(\alpha_{p}t^{p}\right)+\sum_{k=1}^{k=K}\left(\beta_{k}s\left(k,t\right)+\gamma_{k}c\left(k,t\right)\right)\\ \;+\sum_{i=1}^{i=I}\sum_{k=1}^{k=K}\left(\xi_{i,k}t^{i}s\left(k,t\right)+\zeta_{i,k}t^{i}c\left(k,t\right)\right)+\epsilon_{t} \end{array}$$ (4)

where $s(k,t)=\text{sin}(\frac{2\pi kt}{m})$ and $c(k,t)=\text{cos}(\frac{2\pi kt}{m})$. Models of order (P, K, I) ranged between (0, 0, 0) and (1, 2, 1); models for which P < I or K = 0 < I were not considered. The parameters *α*, *β*, *γ*, *ξ*, *ζ* were fit with ordinary least squares. Model selection was performed using the corrected Akaike Information Criterion (AICc); the variance for the normal likelihood component of the AICc calculation was set to the variance of the regression residuals. The detrended time series was taken as the residuals *ϵ*_*t*_ of the model with the smallest AICc value. For the second simulation and the empirical time series, all statistics were estimated from detrended time series.

### Data analysis—Autocorrelation

First-order autocorrelation represents the average relatedness, or correlation, between adjacent observations in a time series. Autocorrelation is typically a value between -1 and 1 (and always is for a first-order stationary process). A time series that results from a first-order autocorrelation process, e.g., *y*_*t*_ = *θ*_1_ × *y*_*t*−1_ + *ϵ*_*t*_, with *θ*_1_ being the first-order autocorrelation coefficient, will not only show a correlation between *y*_*t*_ and *y*_*t*−1_, but also between *y*_*t*_ and observations further back in time than *y*_*t*−1_. These relationships at longer lags appear because non-adjacent observations are indirectly correlated through intermediate direct correlations (e.g., *y*_*t*_ and *y*_*t*−2_ are indirectly correlated because *y*_*t*−1_ is directly correlated with both *y*_*t*_ and *y*_*t*−2_). However, the correlation between *y*_*t*_ and historical *y*_*t*−*k*_ decays exponentially with increasing lag (*k*), with the correlation decaying faster for smaller *θ*(*r*(*k*) = *θ*^*k*^). As a result, time series with large *θ* change slowly and are said to have long-term memory. This relationship between *θ* and the observed rate of change in a time series is a good heuristic for why autocorrelation can be used as an estimate of the return rate of a dynamical system, which in turn indicates its resilience and proximity to a critical transition.

Autocorrelation was estimated at different time scales using two approaches; the first approach involved aggregating time series to different time scales before estimating autocorrelation, and it was also applied to simulated and empirical time series. Time series at different time scales were generated by taking an average of the observations in non-overlapping windows over the entire period of the original time series. Time scales for the simulated series, in units of observations per year, were 24 (the reciprocal is fortnightly, no averaging), 6 (2-mo, averaged groups of 4), 1 (annual, groups of 24), and 0.5 (2-yr, groups of 48). For the empirical time series, averaging was performed to generate time scales, in units of observations per day, of 288 (5-min, no averaging), 24 (hourly, groups of 12), 1 (daily, groups of 288), and 0.5 (2-d, groups of 576). The daily time scale described here corresponds to the averaging procedure used in previous studies of early warning signals in high frequency sensor time series (Batt et al. 2013b, Pace et al. 2017, Wilkinson et al. 2018).

For the first simulation, autocorrelation was estimated from each tier by calculating Pearson’s correlation coefficient between adjacent observations of the time series at each time scale. For the second simulation and empirical time series, autocorrelation was estimated in rolling windows. For the simulation, the window spanned 28-yr and was incremented forward in time by 1 data point before again estimating autocorrelation. For the empirical time series, the window spanned 28-d and was incremented by 6 hours for the sub-daily time scales or by 1 data point for the longer time scales. Each windowed time series was detrended before estimating autocorrelation.

In addition to aggregating, for empirical time series, autocorrelation was estimated at a large number of time scales using the acf function in the *stats* package of the statistical programming language R v3.3.0 [47]. The function can be computed for a full time series to show the correlation between points separated by different amounts of time. In this work, the autocorrelation function was applied to both the simulated and empirical data in 5-min rolling windows for the time series (as above, detrending was performed in each window). Autocorrelation was calculated at time scales between 5 minutes and 2 days at all 5 minute intervals. Because the time series were at a 5-minute resolution, the autocorrelation at a 5-minute time scale is equivalent to first-order or lag-1 autocorrelation.

## Results

**Fig 2:**
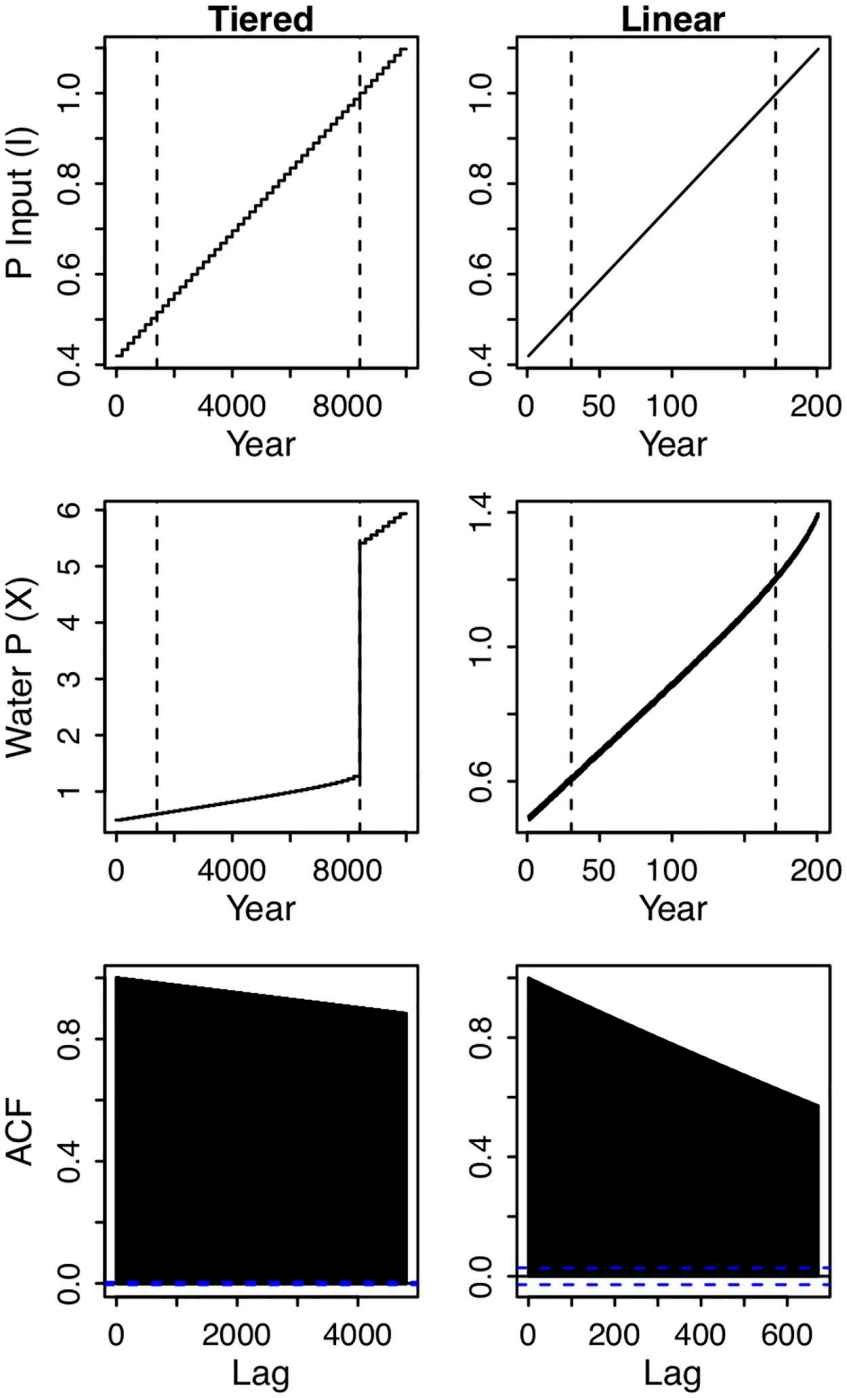
Time series of simulated lake phosphorus. Simulations included 24 observations per year for the entire period of study. The first row shows time series of phosphorus input (*I*), the second row water phosphorus (X), and the third row the autocorrelation function. The left column of panels contains results from a simulation in which *I* was increased in a stepwise fashion; the model was initiated at equilibrium at the start of each tier of P input, each of which lasted 200 years. The maximum number of lags in the bottom-left panel is equal to the number of observations in 1 tier of P input (200*24). The right column of panels contain results from a simulation in which P input is increased smoothly and linearly over time; each time step has slightly higher *I* than the previous. The number of lags in the bottom-right panel are equal to the number of observations in 28 years. The vertical dashed lines indicate the times at which alternate equilibria emerge (a stable node and a saddle point), and when the original equilibrium (a stable node) is annihilated as it collides with a saddle point (see Fig 1). Both simulations are stochastic, but the simulation on the right includes an additional sinusoidal process. The simulation with smooth and linear increases in *I* (right) does not show an abrupt increase in X because the system is lagging behind its equilibrium value, which changes with *I*.

In both simulations, water P increased with P input (Fig 2). In the tiered simulation, the time series underwent an abrupt transition as P input crossed the second critical point. For the simulation with linear, continuous P input, water P never underwent an abrupt transition, despite crossing the critical point in P input. The difference between these two scenarios indicates that the system departed from equilibrium when P input was continuously increased (Fig 2), and that the system can take many years to approach equilibrium after a change in conditions (e.g., trajectories in Fig 1). Both simulations show that autocorrelation is higher between proximal observations than between distant observations (Fig 2), reflecting the time-scale dependence of autocorrelation.

**Fig 3:**
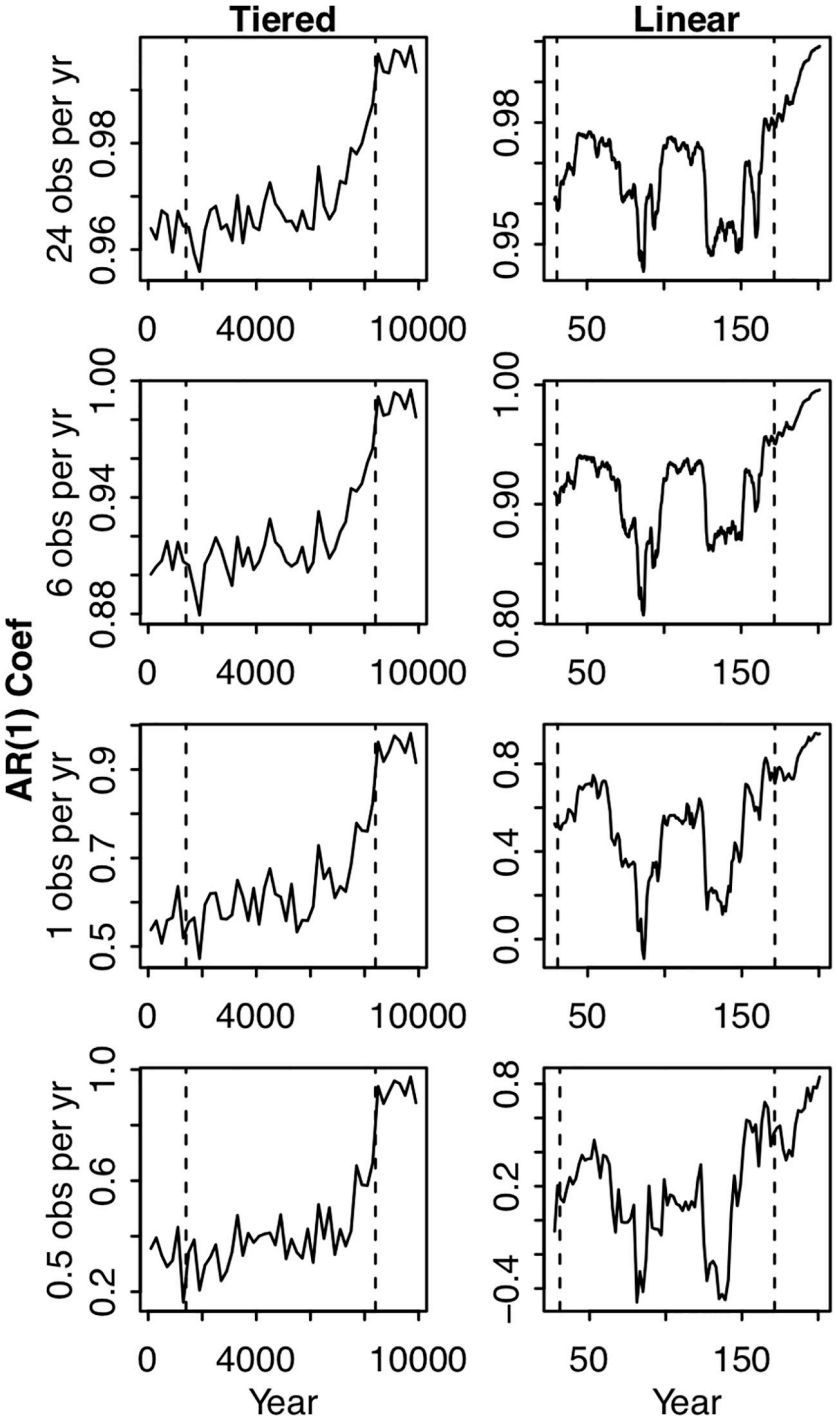
Autocorrelation calculated at different time scales for the two simulated scenarios of tiered increases in P input (left column) and continuous linear increases in P input (right column). For the tiered increases, autocorrelation was calculated within a given tier, never across tiers. For the continuous linear increases, autocorrelation was calculated in a backwards-looking rolling window. Each row of panels corresponds to a different level of pre-analysis aggregation: in the top row no aggregation was performed, in the second row groups of 4 observations were averaged, in the third row the 24 annual observations were averaged, and for the fourth row 2 ‘years’ of observations were averaged. Vertical dashed lines correspond to the critical points described in Figs 1 and 2.

Although autocorrelation is well-known to be time-scale dependent, in the context of early warnings of regime shifts, the dynamics of autocorrelation serve as the indicator. Namely, autocorrelation is expected to increase as a regime shift is approached. In both simulations, autocorrelation increased across the four reference time scales prior to the second critical point (Fig 3). The average value of the autocorrelation time series was higher at shorter time scales (Figs 2 and 3), but the general pattern of increasing autocorrelation was not qualitatively different across time scales (Fig 3). Therefore, in the simulations, increasing autocorrelation was not a scale dependent pattern.

**Fig 4:**
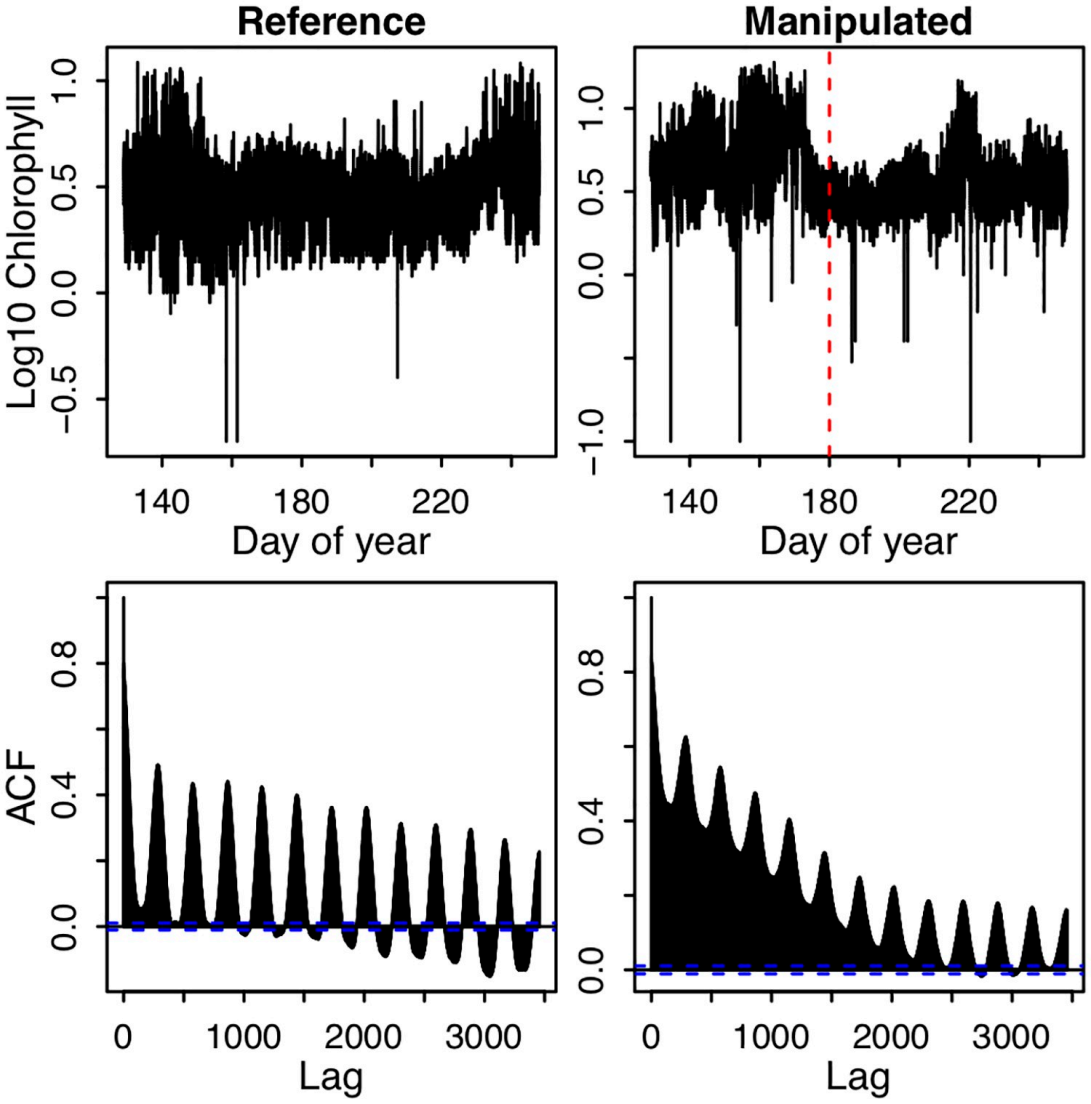
Time series and autocorrelation function of high frequency *in situ* chlorophyll fluorescence measurements in Peter Lake (manipulated) and Paul Lake (reference) in 2015. The red vertical dashed line indicates the day when fertilization was halted in Peter Lake. For the bottom panels, vertical bars are drawn from 0 to a height corresponding to the correlation, and the horizontal axis is the time lag of the correlation measured in time steps for the entire period of study. The horizontal blue dashed lines surround the (relatively narrow) region above or below which the values of autocorrelation are significant at α = 0.05. The time series resolution is five minutes, such that the correlation at a lag of 288 represents the autocorrelation at a daily time scale.

The two lakes differed in the timing and magnitude of peaks in chlorophyll fluorescence, with Paul Lake (reference) showing the highest levels of chlorophyll near the beginning (day 145) and end (240) of the time series, and Peter Lake (manipulated) with a major peak near day 160–170, and secondary peaks near days 140 and 220 (Fig 4). Chlorophyll in Peter Lake was low near day 180, which was the date on which experimental fertilization was halted in response to detected early warning signals.

In addition to the low-frequency peaks in the chlorophyll time series, substantial variability is apparent at much shorter time scales (Fig 4). Part of this variability may be noise, but the autocorrelation function (ACF) of these time series reveals peaks in autocorrelation at lags that are multiples of 288, indicative of a strong diel cycle in the time series (Fig 4). Note that the ACF in Fig 4 was calculated on time series before detrending. Comparing between lakes, chlorophyll ACF is on average much higher in Peter Lake (manipulated) than in Paul Lake (reference). The ACF of both lakes decreases rapidly as lag increases (though, it does peak every 288 points). The cycling and decrease of ACF at higher lags indicate that autocorrelation is dependent on time scale.

**Fig 5:**
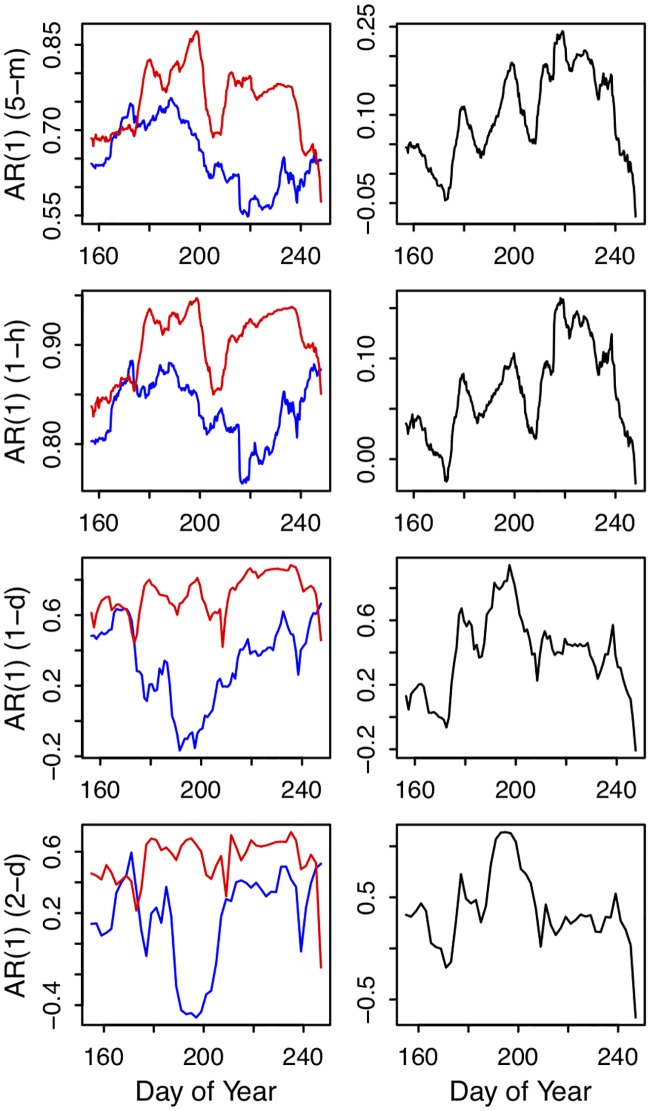
Rolling windows of first-order autocorrelation from detrended chlorophyll time series. Blue lines are from Paul Lake (reference), red lines are Peter Lake (manipulated). In the second column, the black lines represent the difference (Peter—Paul) between the lines in the first column (positive values indicate that autocorrelation was higher in Peter than in Paul). Time series in each row were aggregated to a different time scale prior to analysis (indicated in the y-axis label: 5-minute, 1-hour, 1-day, 2-day).

To test for time-scale dependence of autocorrelation dynamics, we first calculated autocorrelation in a rolling-window format on time series aggregated to four time scales, two sub-daily (5-min, 1-h), daily, and at a 2-d scale. Focusing on the time period leading up to day 180 (when the fertilization was halted), the autocorrelation of the reference lake peaks at all time scales around day 170, and the manipulated begins to rapidly increase around the same time and across all time scales (Fig 5). Between days 170 and 210, the autocorrelation of the manipulated lake is elevated across time scales and is higher than in the reference lake, although the magnitude of this elevation relative to the surrounding time period appears to get smaller as time scale is increased. The difference in autocorrelation between the two lakes shows a sharp increase around day 170 across all time scales. At shorter time scales, the difference in autocorrelation tends to grow until day 235, whereas at longer time scales, the difference in autocorrelation peaks around day 190, stabilizes around a lower value starting around day 210, then drops sharply around day 235.

**Fig 6:**
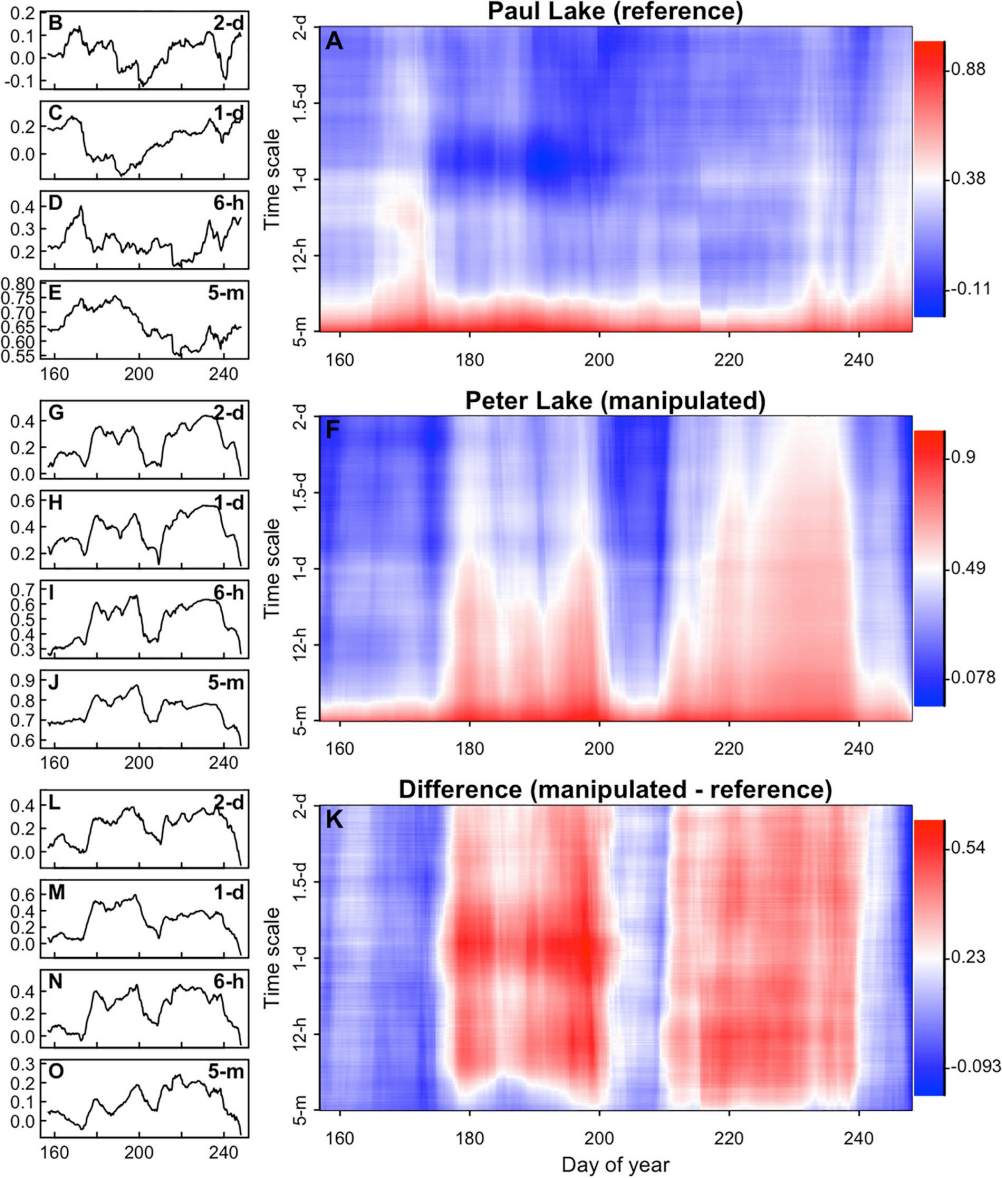
Autocorrelation of chlorophyll over time and across time scales. Panels A,F, and K indicate the autocorrelation (color) of the time series across time scales (vertical axis) through time (horizontal axis). Autocorrelation was computed by calculating the autocorrelation function in a rolling window (see Methods). Panel K is produced by subtracting Panel A from Panel F. Each of the time series panels to the left indicates autocorrelation dynamics at a single time scale. These panels use the same horizontal axis as panels A,F, and K, but represent autocorrelation on the vertical axis instead of as a blue-white-red color gradient.

We also examined the scale-dependence of autocorrelation by calculating the ACF (autocorrelation across all time scales ranging from 5-min to 2 days, in 5-min increments) in rolling windows (Fig 6). As indicated by the ACF for each whole time series (Fig 4), rolling window ACF indicates that at any point in the time series, the highest autocorrelation occurs at shorter time scales (Fig 6A, 6F and 6K). The results from the rolling window ACF are generally similar to the results from AR(1) models applied to different degrees of aggregation: in general, the dynamics of autocorrelation are similar across time scales. For example, autocorrelation in the reference lake is elevated around day 170 and again near day 235 at most time scales (Fig 6A). The intensity of this peak and the subsequent rate of decline vary slightly across time scales (more easily visualized in Fig 6B–6E). In the manipulated lake, autocorrelation is noticeably elevated between days 175 and 200, and again between days 210 and 235. However, the timing and relative size of these peaks varies somewhat across time scales (Fig 6G–6J). With respect to early warning signals, a sharp increase in the between-lake difference in autocorrelation is present around day 175 at across all time scales (Fig 6K–6O).

In general, across all time scales, the temporal changes in autocorrelation were larger in the manipulated lake than in the reference lake. Consequently, the between-lake difference in autocorrelation (Fig 6K–6O) more closely resembles the dynamics in the manipulated lake (Fig 6F–6J) than the dynamics of the reference lake (Fig 6A–6E). The largest departure from this general pattern is that autocorrelation is consistently elevated between days 175 and 200 in the manipulated lake at all time scales, but at short time scales the between-lake difference exhibits two distinct peaks during this period (Fig 6N and 6O). In the first analysis involving aggregation, the between-lake difference in autocorrelation exhibited two peaks between days 175 and 200 at each of the four time scales (Fig 5). However, in both types of analysis and across all time scales, there is a sharp increase in autocorrelation near day 175 in the manipulated lake and in the between-lake difference (Figs 5 and 6).

## Discussion

Translating early warning signals for management is a challenge due to the number of sampling and analytical choices one has to make. For example, most studies of early warning signals have only evaluated statistics at a single time scale, but scale dependence could result in inconsistent conclusions regarding ecosystem resilience and impede application of the approach. Previous analyses of this experiment found early warning signals at a daily time scale prior to day 180 [18, 42]. In this work, we found early warnings in the form of increased autocorrelation leading up to day 180, at a daily time scale and all other time scales ranging between 5-min and 2-d. Furthermore, we found that autocorrelation dynamics were similar across time scales prior to simulated critical transitions.

It is conceivable that our results could have differed if a more extreme range in time scale was analyzed, such as fractions of a second to years. Accordingly, researchers and practitioners using early warning signals should be aware of this possibility and understand that choosing too coarse a time scale could prevent the relevant dynamics from being resolved; similarly, a time scale too fine could introduce noise from processes unrelated to the critical transition. For example, in their study of a model of thermohaline circulation, Held and Kleinen [10] aggregated data into 50-year time steps, describing variation at shorter time scales as “weather noise” that might contaminate the autocorrelation signal. Models of algal blooms involve interactions among nutrients, phytoplankton, and zooplankton [39, 41]. As a starting point for the relevant time scale of this dynamical system, one might consider generation time as a factor in how fast a plankton system changes, and therefore at what time scale it should be analyzed. Phytoplankton generation times are estimated to be between several hours to multiple days [48], and zooplankton generation times vary between days and weeks [49]. Therefore, ecological intuition would suggest that a time scale of hours might be appropriate. By contrast, the model of water and sediment P uses parameter values grounded in field observations [35] and proceeds in annual time steps, and can take many decades to approach equilibrium. In this case an hourly analysis seems unnecessary, and data collection and analysis at the scale of weeks to years more appropriate. To avoid gross over- or undersampling, some *a priori* knowledge of the system’s general behavior may be helpful.

In addition to ecological or system-specific expertise, there are also empirical, data-driven approaches to identifying appropriate time scales. In some cases, the characteristic time scale of a time series may be defined as the first nadir of the autocorrelation function, or when the autocorrelation function first crosses 0. For the chlorophyll time series in these lakes, these would correspond to 12 hours and 6.5 days, respectively (Fig 4), which roughly correspond to the time scales at which early warning signals were found in this study and in previous analyses [18, 42]. Practitioners should be able to identify a suitable time scale for analysis of early warnings because autocorrelation dynamics are similar within a substantial margin of a nominal scale, which might be identified through means such as domain (ecological) expertise or empirical time series analysis.

The issue of time scale is just one of many practical challenges to detecting early warnings signals of critical transitions. Much progress has been made with respect to determining which statistics best capture the phenomenon of critical slowing down [23, 24], and evaluating potential differences in the performance of these statistics across different types of critical transitions [19]. Another challenge facing the application of early warning signals is assessing the risk of false positives or negatives when interpreting early warning statistics, especially with regard to *a priori* knowledge that the ecosystem is experiencing a critical transition [15, 20, 22, 50]. Variable selection is also critically important because although a full suite of variables could possibly be measured in an ecosystem, only a fraction of these are typically related to the critical transition at hand; hence, unrelated variables would not be expected to exhibit critical slowing down. For example, variables like salinity are not closely tied to an algal bloom, and other variables might be too noisy or too heavily influenced by other processes to yield early warning signals [26]. Accordingly, results from many variables, statistics, or time scales can present an analyst with conflicting impressions of whether or not early warning signals are present [16]. Some researchers have confronted this problem by using dimension reduction techniques [10, 13, 16, 43, 51], but these methods may be influenced by unrelated processes and patterns. Researchers recognize that complex system drivers are often unknown [52, 53], promoting interest in developing and using methods that can analyze trends in multiple variables simultaneously. For instance, Litzow et al [54] aimed to detect a critical transition in marine community dynamics by assessing variance in fish catch data. They found that while assessing trends in individual fisheries were not statistically significant, pooling multiple populations aided in the detection of the collapse. If early warning theory is to be a useful tool for monitoring changes in ecosystem resilience near a critical transition, more research is needed on how to handle multidimensional inputs, especially if patterns are inconsistent across these dimensions. Multivariate approaches like the variance index, Fisher information and multivariate time series modelling have shown promise (e.g., [16, 43, 55]), but more work is needed to test the robustness of these methods.

The consistency of autocorrelation dynamics across time scales highlights the potential of the indicator and suggests that detecting early warning signals may not be dependent on determining which time scale is most reliable. The result also implies that current monitoring efforts may be sufficient for detecting these statistical signals, particularly in cases where the data volume during the approach of a transition is large enough to estimate statistical indicators like autocorrelation. Furthermore, since the problem of data acquisition is diminishing with the expansion of sensor technology, large data sets and reliably computed warning indicators can help prepare for, and possibly avoid, algal blooms and other problematic critical transitions.

Here, we provided evidence that the dynamics of lag-1 autocorrelation are robust to the time scale of the analysis for the case studies presented. The cross-scale consistency was especially strong near the critical transitions in a simulation and a field experiment, in which autocorrelation increased at all time scales as the transition was approached. For early warning signals to be a useful management tool, decisions on computational techniques or interpretation cannot be both arbitrary and critical to the outcome of the analysis. Managers and stakeholders have a need for scientists to translate the results (like those presented in this paper) into guidance for ecosystem management. While more research is needed in the area of early warning signals, this work offers insight regarding the impact of timescale on a prominent measure of critical slowing down. Follow-up studies should be conducted to test the universality of this discovery, but the findings in this paper have great potential to improve ecosystem management, moving forward.
